# Population-Level Associations between Preschool Vulnerability and Grade-Four Basic Skills

**DOI:** 10.1371/journal.pone.0007692

**Published:** 2009-11-25

**Authors:** Amedeo D'Angiulli, William Warburton, Susan Dahinten, Clyde Hertzman

**Affiliations:** 1 Institute of Interdisciplinary Studies, Carleton University, Ottawa, Canada; 2 Human Early Learning Partnership, The University of British Columbia, Vancouver, Canada; 3 Department of Nursing, The University of British Columbia, Vancouver, Canada; 4 Department of Health Care and Epidemiology, The University of British Columbia, Vancouver, Canada; University of Granada, Spain

## Abstract

**Background:**

This is a predictive validity study examining the extent to which developmental vulnerability at kindergarten entry (as measured by the Early Development Instrument, *EDI*) is associated with children's basic skills in 4th grade (as measured by the Foundation Skills Assessment, *FSA*).

**Methodology/Principal Findings:**

Relative risk analysis was performed on a large database linking individual-level EDI ratings to the scores the same children obtained on a provincial assessment of academic skills (FSA – Foundation Skills Assessment) four years later. We found that early vulnerability in kindergarten is associated with the basic skills that underlie populations of children's academic achievement in reading, writing and math, indicating that the Early Development Instrument permits to predict achievement-related skills four years in advance.

**Conclusions/Significance:**

The EDI can be used to predict children's educational trends at the population level and can help select early prevention and intervention programs targeting pre-school populations at minimum cost.

## Introduction

There is a general consensus among social scientists of the inestimable social, economic and global value of monitoring children's educational trends at the group (i.e., school district) or population (i.e., province, state) level [1]–[2]. Early child development outcomes are not only markers of child well-being, but are also important predictors of the child's potential for productive employment, and integration into adult society [3]. Additionally, there is accumulating evidence of the importance of early development for healthy aging and on the later development of a range of chronic diseases [4]. However, there has been no stringent evidence that the developmental and educational pathways of populations of children can be forecasted early enough and reliably [5], let alone at reasonable costs.

In the present study, we report evidence that early developmental vulnerability in kindergarten –as reflected by the Early Development Instrument (EDI, [6]) – is associated with the basic skills assumed to underlie children's academic achievement. Our findings indicate that, on the basis of the EDI, achievement-related basic skills can be statistically predicted at least four years in advance and with an unusual level of precision.

The scales of the EDI are deemed to map onto domains of early child development involving physical, social-emotional, and language/cognitive milestones that have lifelong influence on health, well-being, behaviour and learning skills [7]–[8]. Because these domains are all interconnected with optimal brain development and maturation, it has been argued that the EDI can be considered a reasonable proxy for measuring early optimal brain development at population level [9]–[10]. Although the notion of optimal brain development captures the essence of early development science, the acceptance, adoption and use of such broad and encompassing concept at a population level depends on researchers in several different fields adopting the shared assumption that the early development of groups of children are related to the competence, coping skills, and health of populations throughout life. Until that common ground is achieved, *readiness for school* (or *school readiness*) as reflected by the EDI may be used as a proxy measure for optimal brain development.

As noted by Mustard and Young [10], both readiness for school and the related, albeit different, notion of *readiness to learn* are widely used concepts in education and policy sciences [6]. Although neither exhaust the full implications of brain development during the first years of life and its critical links to human development, measuring school readiness through the EDI is promising for practical reasons, since entry into school is the first time after early childhood when all children are enrolled in an institutional structure where population-level data on children's development can be collected efficiently and at reasonable costs. In addition, the EDI can serve as a common framework for defining early child development in a comprehensive fashion in terms of early group or population-level determinants of children's performance in the school system.

Being a measure of children's overall development, the EDI should provide information about populations of children that can be interpreted forwards in time, offering an indication of their future basic skills performance. On this hypothesis, we tested whether the incidence of vulnerability on EDI scores obtained at kindergarten entry from a representative sample of the population of children from British Columbia could predict their not meeting expectations on a provincial assessment of academic skills (FSA – Foundation Skills Assessment) years later, when they had almost completed grade 4.

## Materials and Methods

### Measures

#### Early Developmental Instrument

In British Columbia, the transition period from preschool to school is called Kindergarten; it begins in September of the year the child turns five years old. In February of the Kindergarten year (after approximately six months of knowing their students), teachers complete the EDI for all of the children in their classrooms.

The EDI combines domains that have been identified as relevant to children's readiness for school [7], [11]–[13]: 1) physical health and well-being; 2) social knowledge and competence; 3) emotional health and maturity; 4) language and cognitive development; 5) communication skills and general knowledge.

The three primary purposes of the EDI are to: (1) report on populations of children in different communities, (2) monitor populations of children over time, and (3) predict how groups of children will do in elementary school, both academically and socially. Designed to take 20 minutes per child to complete, the EDI consists of 104 questions designed to tap five scales of early childhood development [14]. These five scales include: 1) *physical health and well-being*. The physical scale includes items that assess children's gross and fine motor skills, pencil holding, running on the playground, motor coordination, energy levels for classroom activities, independence in looking after own needs, and daily living skills; 2) *social knowledge and competence*. The social scale includes items about children's curiosity about the world, eagerness to try new experiences, knowledge of standards of acceptable behaviour in a public place, ability to control own behaviour, appropriate respect for adult authority, cooperation with others, following rules, and ability to play and work with other children; 3) e*motional health and maturity*. The emotional scale assesses children's abilities to reflect before acting, balance between too fearful and too impulsive, abilities to deal with feelings at age-appropriate levels, and empathic responses to other people's feelings; 4) *language and cognitive development*. The language scale includes items designed to tap children's reading awareness, age-appropriate reading and writing skills, age-appropriate numeracy skills, board game performance, abilities to understand similarities and differences, and ability to recite back specific pieces of information from memory; 5) *communication skills and general knowledge*. The latter scale includes items that assess children's skills to communicate needs and wants in socially appropriate ways, symbolic uses of language, storytelling, and age-appropriate knowledge about the life and world around them.

The EDI has been validated on several thousands of children across Canada and Australia [14]–[16]. In addition, the instrument includes versions that implement recommendations to extend its validity to subgroups of children coming from minorities [17]–[18].

The principal parameter generated from each scale of the EDI is ‘vulnerability’. Each child's EDI is divided into the items of a specific scale and scored between 0 and 10. A ‘10’ means that the child is doing all the things he/she should be doing developmentally, all of the time, at kindergarten age; whereas a score of 0 means the child is doing none of them ever. Children's domain-specific scores are converted into a dichotomous measure of vulnerability based on whether or not the score falls below a domain-specific cut-off score equal to the bottom 10% of average percent rate in each scale for the province, based on the first cycle of data collected in British Columbia. Thus, the child may be found to be “vulnerable” on 0 to 5 measures of pre-school development (henceforth, referred to as *number of EDI vulnerabilities*).

It is important to specify that the present approach does not simply mean that 10% of kindergarten children will by definition fall into the “vulnerable” category because the cut off is referred to a distribution of average scores from 59 school districts across the entire province. Because of the large variation in the rates of vulnerabilities reported across the school districts, determining whether an average rate obtained from a particular group of children is representative of the population can only be done by assessing whether this average falls within the 95% confidence interval for the entire distribution of average rates across the province. All the properties of this distribution are known and fully documented [19] (and publicly available: ecdportal.help.ubc.ca), thus, to better understand the analysis that we will present later, it is useful to note down that the 95% confidence intervals for provincial vulnerability rates were: 5.03%–18.26% for physical health and well-being, 5.03%–17.50% for social knowledge and competence, 5.57%–15.79% for emotional health and maturity, 3.30%–16.40% for language and cognitive development, 2.78%–22.04% for communication skills and general knowledge.

#### Foundation Skills Assessment

Similar to the EDI, the FSA is administered “universally” in BC to directly test the reading, numeracy, and writing skills of children in grades 4 and 7. The term “universally” is used here as in Offord [2]. That is, although both the EDI screening and the FSA assessment ‘programs’ are aimed at the general school population and potentially open to every Kindergarten and Grade 4 student, this does not mean that each and every student receives the assessment, only that the assessments are not targeted to a specific group.

The results of the FSAs are partitioned in three categories of children: (a) those performing below expectations, (b) those meeting expectations and (c) those exceeding expectations relative to other same-grade peers in the province. The cut-off scores for these categories are based on performance standards developed by BC Ministry of Education [20]–[22]. In the present analysis, we collapsed categories b and c into a single category called *above expectations*, since the focus of this particular study was just on predicting a pass/fail outcome – having vs. not having minimum appropriate basic skills.

### Linkage Procedure

The archival data used for the present analysis were compiled through unique anonymous probabilistic linkage between EDI and FSA records retrieved from Edudata Canada, an education research centre that houses the student level Ministry of Education data for all BC children, kindergarten to Grade 12. The data that we used is available for research that has undergone rigorous ethical review and meets required methodological and reporting quality standards at institutions and universities in Canada. Details regarding the application process for data from the Ministry of Education can be obtained from Edudata Canada. (http://www.edudata.educ.ubc.ca/researchers/researcher_help.htm). Details regarding application for data from the EDI can be obtained from the Human Early Learning partnership. (http://www.earlylearning.ubc.ca/privacy-faq.html#6).

Children's EDI records were first linked with their Ministry of Education Provincial Education Numbers (PENs) through a combination of gender, date of birth, and home postal code flags. The combination of birth date and postal code is excellent for matching in a jurisdiction the size of BC. When the EDI records were collected, the population of BC was very close to 4 million. The median number of people with each postal code in BC was about 100. The median number of people with each birth date in BC was about 150. If birth dates and postal codes were distributed randomly, the probability of 2 people sharing the same postal code and birth date would be vanishingly small. We note that the Fellegi-Sunter method of probabilistic linkage is not appropriate here because the distribution of postal codes and birth dates is known to be relatively constant. (We did not use a weighted match procedure either – weights are very useful when matching on name since some names are exceedingly more common than others – our combination of identifiers bypassed this critical confound as well.) Therefore, we used an absolute threshold for establishing a link, in that we accepted a pair of records as a link if the combination of postal code and birth date was exact and unique.

In spite of this optimal record linking context, there were still two caveats. First, all individuals in small towns shared the same postal code. Second, twins shared both postal code and birth date. Empirically, however, these problems only introduced a small amount of uncertainty, as shown by our checking procedures. In a different sample, we checked individual matching errors between personal education number as well as birth date and postal code. Only 0.8% of the records that were defined as a match based on birth date and postal code did not match on personal education number. Because there will be some data entry error in personal education numbers, this represents an upper bound for the error rate. In sum, it is reasonable to conclude that in a jurisdiction with the characteristics and size of BC, postal code and birth date were sufficient variables for linkage, any residual problems was caused by data entry errors.

### Database and Preliminary Analysis

The PEN is a permanent number used throughout the kindergarten to grade 12 education system in British Columbia. The PEN was then used to access FSA scores and successively link them with the child's EDI scores. Linkage rates were very high at both stages. PENs were identified for 70% of children with EDI scores. Thus, there were 7,910 distinct EDI identification numbers from the following school districts: Abbotsford, Vancouver, Coquitlam and Howe Sound. Their EDI information was collected in school years 1999/2000 and 2000/2001. The mean age of the students was 5.7 years (SD = 0.3) at the time of the EDI, and 9.7 years (SD = 0.3) at the time of the FSA.

The selection of this four districts (out of 59) was based on the availability of both EDI and FSA (i.e., the latter was collected in school years 2003/2004 and 2004/2005) at the time when the analysis was undertaken. Because school and year were known, and because the EDI was virtually universal, the number of Education records that we searched for matches was then only slightly higher than the possible matching links. As expected, when linking EDI and grade 4 FSA, we obtained 6838 matched records for a success linkage rate of 86.4%, which indicated the number and percentage of children who attended grade 4 in the mentioned public school districts and who possessed records for both EDI and FSA. The main source of missed matching was omitted birth days in some EDI records. Missing information on some variables meant that not all of these records were used on all of the analyses but this reduced our sample negligibly (sample sizes for each variable are reported in the Table below).

### Relative Risk Analysis

We used relative risk (or risk ratio: RR) to estimate the magnitude of association between types of early developmental vulnerabilities and not having minimum appropriate basic skills in achievement-related areas (i.e., meeting expectations for FSA performance); this expresses the likelihood of not acquiring basic skills in groups vulnerable on the EDI relative to non-vulnerable control groups. In this study, the RR corresponds to the ratio of the odds of vulnerability among the cases to that among the controls (*ad*/*bc*, where *a* = EDI-vulnerable cases meeting basic skills expectations, *b* = EDI-vulnerable cases not meeting expectations; c = EDI-non-vulnerable cases not meeting expectations, *d* = EDI-non-vulnerable cases meeting expectations).

A relative risk of 1.0 indicates that the risk of not meeting expectations in the vulnerable and non vulnerable groups are the same and that therefore there is no association between early vulnerability and not acquiring basic skills. However, a value greater than 1.0 indicates a positive association, or an increased risk among those vulnerable in one of the EDI domains (for example, an RR of 1.4 would indicate that children vulnerable on the EDI in a particular domain have 1.4 times the risk or are 40% more likely not to meet expectations).

## Results and Discussion

Our analysis shows that groups of children vulnerable on any one of the EDI scales are more likely to perform below expectation on FSA scores in all academic areas in grade 4 (see Table 1), suggesting that in groups of children the EDI is associated with and predicts basic skills performance four-years after kindergarten; the narrow confidence-interval function reveals high precision of these estimates. Our risk ratios show that children with early developmental vulnerabilities are about 2 to 4 times more likely to score below expectations in the FSA.

**Table 1:** Case counts, percentages, Relative Risk (RR) and 95% Confidence Interval ([CI]) of ‘Failing to Meet Expectations’ on Foundation Skills Assessments (FSA) in Grade 4 for Children ‘Vulnerable’ on the Early Development Instrument (EDI) in Kindergarten.

	Performance on FSA			
	Below expectation	Above expectation	Total	RR [CI]
EDI Vulnerability				
*Physical*	(Numeracy)			
Yes	356 (5.23)	580 (8.51)	936 (13.74)	
No	872 (12.80)	5004 (73.46)	5876 (86.26)	
Total	1228 (18.03)	5584 (81.97)	6812 (100.00)	
				2.56 [2.31–2.84]
	(Reading)			
Yes	440 (6.46)	496 (7.28)	936 (13.74)	
No	1273 (18.69)	4603 (67.57)	5876 (86.26)	
Total	1713 (25.15)	5099 (74.85)	6812 (100.00)	
				2.16 [1.99–2.36]
	(Writing)			
Yes	310 (4.55)	626 (9.19)	936 (13.74)	
No	664 (9.74)	5212 (76.52)	5876 (86.26)	
Total	974 (14.29)	5838 (85.71)	6812 (100.00)	
				2.90 [2.61–3.29]
*Social*	(Numeracy)			
Yes	282 (4.14)	417 (6.13)	699 (10.27)	
No	942 (13.84)	5164 (75.89)	6106 (89.73)	
Total	1224 (17.99)	5581 (82.01)	6805 (100.00)	
				2.61 [2.35–2.91]
	(Reading)			
Yes	332 (4.88)	367 (5.39)	699 (10.27)	
No	1377 (20.24)	4729 (69.49)	6106 (89.73)	
Total	1709 (25.11)	5096 (74.89)	6805 (100.00)	
				2.10 [1.92–2.31]
	(Writing)			
Yes	250 (3.68)	449 (6.59)	699 (10.27)	
No	721 (10.59)	5385 (79.14)	6106 (89.73)	
Total	971 (14.27)	5834 (85.73)	6805 (100.00)	
				3.02 [2.68–3.42]
Emotional	(Numeracy)			
Yes	273 (4.04)	493 (9.30)	766 (11.34)	
No	941 (13.93)	5047 (74.73)	5988 (88.66)	
Total	1214 (17.97)	5540 (82.03)	6754 (100.00)	
				2.26 [2.03–2.54]
	(Reading)			
Yes	324 (4.80)	442 (6.54)	766 (11.34)	
No	1374 (20.34)	4614 (68.52)	5988 (88.66)	
Total	1698 (25.14)	5056 (74.86)	6754 (100.00)	
				1.84 [1.68–2.03]
	(Writing)			
Yes	239 (3.53)	527 (7.81)	766 (11.34)	
No	725 (10.73)	5263 (77.93)	5988 (88.66)	
Total	964 (14.27)	5790 (85.73)	6754 (100.00)	
				2.57 [2.27–2.92]
Language/Cognition	(Numeracy)			
Yes	327 (4.85)	353 (5.24)	680 (10.09)	
No	880 (13.06)	5180 (76.85)	6060 (89.91)	
Total	1207 (17.91)	5533 (82.09)	6740 (100.00)	
				3.31 [2.99–3.66]
	(Reading)			
Yes	432 (6.41)	248 (3.68)	680 (10.09)	
No	1250 (18.55)	4810 (71.36)	6060 (89.91)	
Total	1682 (24.96)	5058 (75.04)	6740 (100.00)	
				3.07 [2.86–3.32]
	(Writing)			
Yes	291 (4.31)	389 (5.78)	680 (10.09)	
No	669 (10.37)	5391 (79.54)	6060 (89.91)	
Total	960 (14.24)	5780 (85.76)	6740 (100.00)	
				3.87 [3.46–4.34]
Communication/Knowledge				
	(Numeracy)			
Yes	344 (5.05)	673 (9.88)	1017 (14.93)	
No	883 (12.96)	4914 (72.11)	5797 (85.07)	
Total	1227 (18.01)	5587 (72.11)	6814 (100.00)	
				2.20 [1.99–2.47]
	(Reading)			
Yes	505 (7.41)	512 (7.52)	1017 (14.93)	
No	1208 (17.73)	4589 (67.34)	5797 (85.07)	
Total	1713 (25.14)	5101 (74.86)	6814 (100.00)	
				2.38 [2.00–2.58]
	(Writing)			
Yes	279 (4.09)	738 (10.84)	1017 (14.93)	
No	693 (10.17)	5104 (74.90)	5797 (85.07)	
Total	972 (14.26)	5842 (85.74)	6814 (100.00)	
				2.29 [2.03–2.59]

*Note.* Values in parenthesis represent percentages. [R4] *physical* is an abbreviation for physical health and well-being domain; *social* is an abbreviation for social knowledge and competence domain; *emotional* is an abbreviation for emotional health and maturity domain; *language/cognition* is an abbreviation for language and cognitive development domain; *communication/knowledge* is an abbreviation for communication skills and general knowledge domain.

For all risk ratios *χ* ^2^(1)≥135.13; *p*<.00001.

Consistent with other research findings showing that levels of early development of language skills predicts levels of achievement in elementary school [23]–[24], language and cognitive development vulnerability on the EDI showed the strongest relationship with all three FSA scores.

**Figure 1:**
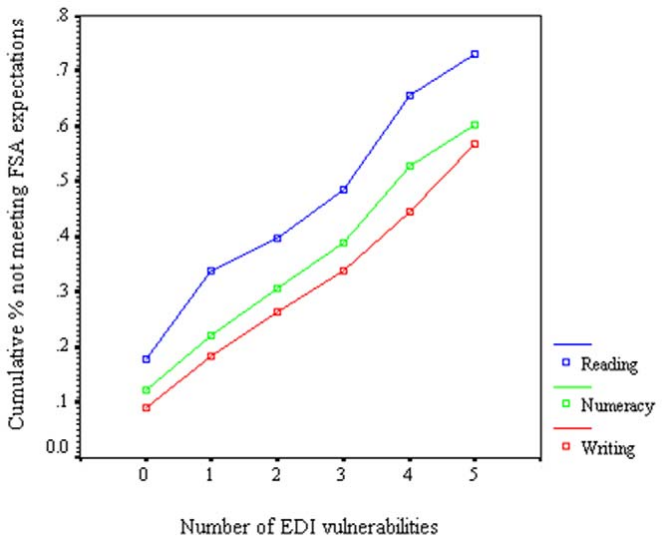
The relationship between cumulating risk (number of vulnerabilities) in kindergarten and the percentage of children failing to meet expectations in grade 4. Note. Orthogonal polynomial linear contrasts for proportions [32] are summarized with the standard normal deviate statistic (*Z*_Reading_ = 43.67, *Z*_Numeracy_ = 33.50, *Z*_Writing_ = 21.76, all *SE*'s<.025, all *P*'s at least<.0001, *two-tailed*).

In addition, the cumulative percentage of children who do not meet expectations increases linearly with numbers of accrued vulnerabilities, i.e., falling below cut-off on one or more EDI scale (see Figure 1), indicating that the trend of risk is consistent with a *linear* cumulative pathway model [25].

The finding that language/cognitive vulnerability yielded the strongest associations may also be explained by the fact that this measure of vulnerability is conceptually closest to the type of literacy basic skills measured by the FSA. On the other hand, the relative risk ratios for language/cognitive vulnerability relative to numeracy are comparable to those for reading and writing, showing the important association between literacy development and numeracy [26]. That vulnerability in the social and emotional areas is a significant predictor is consistent with other studies that have found a relationship between social, emotional and behavioural development, e.g., self-regulation, interpersonal skills with later academic achievement [27]–[28]. The role of physical health has received considerably less attention in educational research, but it has been recognized as a plausible additional barrier to educational achievement in elementary school [29]. Together, the present findings show why a comprehensive measure of early development like the EDI has high predictive value – although the most shared construct yielded the strongest associations, the supplementary associations of other domains of development are also important.

Although in Table 1 the percentage of children identified as Yes for some of the vulnerabilities is over 10%, all percentage rates are within the confidence intervals for the EDI scales distributions in the entire province (see confidence intervals mentioned earlier, pp. 8–9). Therefore, it is possible to conclude that the groups sampled in this study were indeed representative of the population of children of British Columbia.

What are the important practical implications of the EDI for the early learning and developmental sciences? A chief implication is related to the fact that the EDI can be interpreted forwards in time and can help to detect if particular groups of children are at risk once they enter kindergarten. That is, the EDI is a powerful tool for early assessment of school readiness at a population level and a strong predictor of how groups of children will adjust to school.

A complementary implication is that the EDI can also be interpreted backwards in time. That is, it can be used to understand the qualities of early experience that certain groups of children have had up to kindergarten entry. This information may be critical in designing and/or implementing effective preschool compensatory education in promoting and augmenting school readiness. Our findings reinforce the importance of focusing on language development. In particular, because there is evidence that the differential experience of spoken (receptive) language content in vulnerable groups of young children is likely to contribute to the highest risk of poor achievement outcomes later on [30]–[31], addressing population differentials in receptive language should be a priority for early intervention programs. In addition to the urgency of fostering optimal early language acquisition, our results show the importance of expanding early intervention programs to include curricula that directly address social and emotional developmental factors that, in turn, may influence basic linguistic/cognitive abilities and subsequent achievement in early grades and beyond [32].

Finally, it is important to note that although there is a relatively wide recognition of the benefits of linking data from multiple sources (social services, health records, preschool enrollment, school assessments, etc.), the methods to implement multiple, reliable large-scale linkages are very complex and present formidable challenges. Developing unique linkable student identifiers that are consistent across multiple data collection efforts is essential for improving the accuracy and efficiency of conducting large scale analysis. Indeed, analyses like the present one would not be possible without “system-wide identifiers”. In Canada, where the process of implementing provincially and nationally linked databases is proceeding through an experience of at least a decade, system-wide identifiers have gained recognition as the most indispensable tool for interfacing population-level interdisciplinary research, especially at the crossroads between education, health and social policy.

In conclusion, the EDI can reliably predict achievement-related basic skills of populations of children at least four years in advance. This instrument can help select early prevention and intervention programs targeting groups of pre-school children because an entire kindergarten class can be realistically assessed for the cost of a one-day teacher buy-out. For its incalculable return to society, assessing every kindergarten child with the EDI would appear quite affordable.
